# A Case of Right Ventricular Thrombus in a Patient With Recent COVID-19 Infection

**DOI:** 10.7759/cureus.25150

**Published:** 2022-05-19

**Authors:** Andreas Mitsis, Aggelos Alexi, Thrasos Constantinides, Grigorios Chatzantonis, Panayiotis Avraamides

**Affiliations:** 1 Cardiology, Nicosia General Hospital, Nicosia, CYP; 2 Cardiac MRI Department, Nicosia Heart Center, Nicosia, CYP; 3 Cardiology, Athens Medical Group, Athens, GRC

**Keywords:** cardiac magnetic resonance (cmr), intracardiac thrombosis, ventricular thrombosis, right ventricular thrombus, coronavirus disease 2019 (covid-19)

## Abstract

Coronavirus disease 2019 (COVID-19) is a viral respiratory disease caused by severe acute respiratory syndrome coronavirus 2 (SARS-CoV-2). The respiratory system is the main target of the virus; however, apart from lung disease, a relatively large proportion of patients develop thrombosis as well. We present the case of a 19-year-old male who was admitted after contracting community-acquired right-sided pneumonia. The patient had a history of COVID-19 infection four weeks before admission. The echocardiographic assessment revealed a 16 x 6-mm right ventricular (RV) thrombus. He underwent a cardiovascular magnetic resonance (CMR) study, which confirmed the findings. After ruling out the most common causes of hypercoagulability, COVID-19 was judged to be the cause of the thrombus. The patient was treated with warfarin. Follow-up imaging with echocardiography and CMR six months later revealed complete resolution of the thrombus. Hypercoagulability is a major complication of COVID-19 and in situ thrombosis can occur both in the arterial and venous circulation. The recognition of intracardiac thrombi even in low-risk patients with a history of COVID-19 infection and the immediate initiation of antithrombotic treatment to minimize the risk of embolization is of paramount importance. Advanced imaging techniques are often required to establish the diagnosis of this condition.

## Introduction

Even though coronavirus disease 2019 (COVID-19) is primarily a respiratory disease, there is strong evidence to indicate the development of a prothrombotic environment leading to both arterial and venous thrombosis in many infected patients [1]. We present a case of a young male patient with a history of recent COVID-19 infection and a right ventricle (RV) in situ thrombus. After ruling out the most common causes of hypercoagulability, the previous COVID-19 infection was judged to be the cause of thrombus formation.

## Case presentation

A 19-year-old male patient was referred to our hospital after being diagnosed with right-sided pneumonia. He had been admitted two days earlier to a district hospital with community-acquired pneumonia. Upon admission, his polymerase chain reaction (PCR) test had been negative for COVID-19 infection. His past medical history included a history of mild bronchial asthma and a recent COVID-19 infection four weeks ago. For that, he had not required hospitalization and only received symptomatic treatment with inhalers at home.

**Figure 1:**
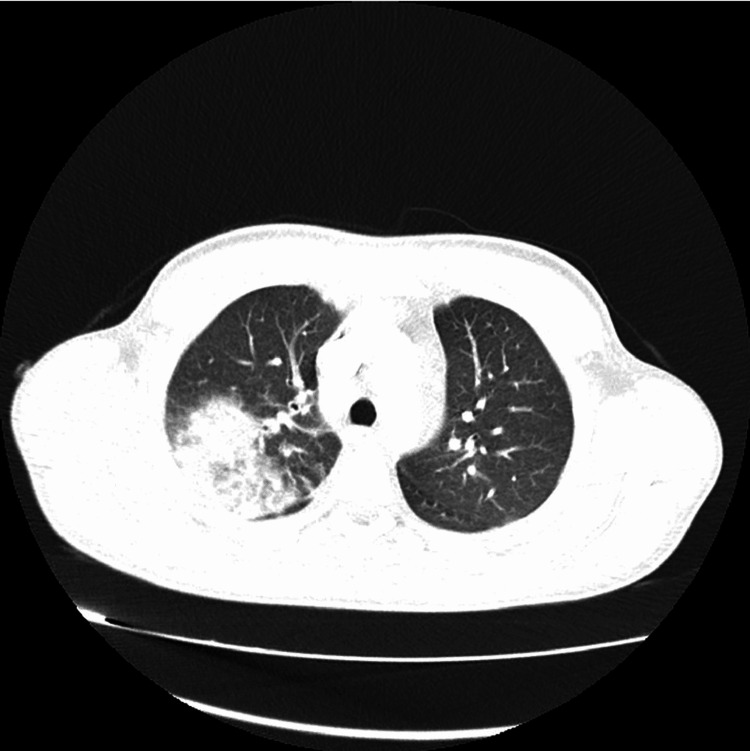
CT lung CT lung showing right-sided pneumonia and right-sided pleural effusion CT: computed tomography

Upon admission to our department, the patient was febrile (temperature of 39 ^o^C) and hemodynamically unstable. Vital signs showed tachycardia at 117 beats per minute and decreased blood pressure at 90/66 mmHg. SO_2_ was 94% in a 28% Venturi mask, with a respiratory rate of 16 breaths per minute. Physical examination revealed right-sided rales. There were no signs of left ventricular (LV) failure nor any evidence of fluid retention. A chest X-ray revealed a normal cardiac silhouette and evidence of right-sided pneumonia. An electrocardiogram showed sinus tachycardia without evidence of ischemia. Laboratory workup revealed an elevated C-reactive protein at 381 mg/l, elevated d-dimers at 3,661 ng/ml, a normal high sensitivity troponin I of 0.11 ng/L, an elevated pro-brain natriuretic peptide of 1,000 pg/mL, and elevated white blood cells of 13,500/μl with prominent neutrophilia (94.4%). A urine antigen test for Legionella pneumophila, Mycoplasma pneumoniae, and Streptococcus pneumoniae did not show culture positivity. Blood cultures were also negative. A CT pulmonary angiogram ruled out pulmonary embolism and revealed right-sided pneumonia with right-sided pleural effusion (Figure 1). A repeat COVID-19 PCR test was negative.

**Figure 2:**
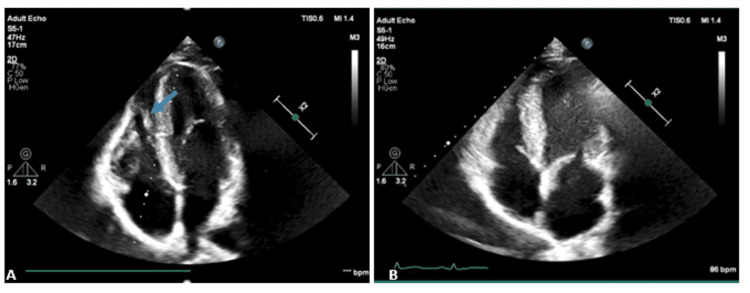
Transthoracic echocardiogram in the acute phase and after six months 2A. Transthoracic echocardiogram in four-chamber view revealing an oval-shaped mass (blue arrow) in the apex of the right ventricle 2B. Follow-up transthoracic echocardiogram after six months of adequate anticoagulation showing complete resolution of the thrombus

Transthoracic echocardiography (TTE) showed a normal LV size and function, a normal RV size and function, and a mass at the apex of the RV (Figure 2A). The patient was admitted to the cardiology critical care unit, and low-molecular-weight heparin (LMWH) was started. IV fluids were administered, and an IV antibiotic treatment with meropenem and vancomycin was also initiated. The patient responded well to the IV fluids and his hemodynamic status quickly stabilized. Over the following days, the inflammation markers were normalized too. The follow-up TTE confirmed the initial findings and the suspicion of the RV mass. A vein Doppler ruled out deep vein thrombosis.

**Figure 3:**
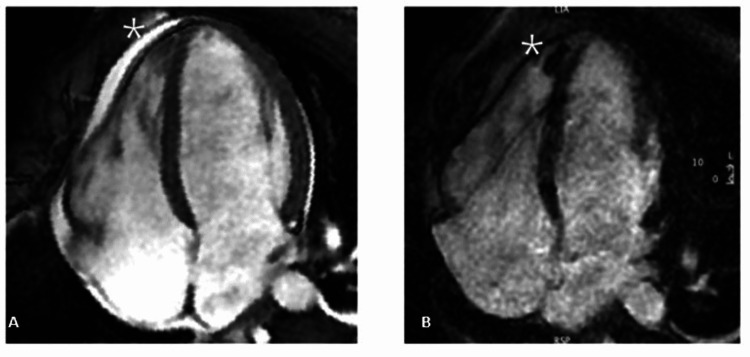
Cine balanced steady-state free precession (bSSFP) and late gadolinium enhancement (LGE) imaging 3A. Cine balanced steady-state free precession (bSSFP) imaging reveals a mass (white star) within the cavity of the right ventricle apex, with borders distinguishable from ventricular endothelium and trabeculation 3B: Late gadolinium enhancement (LGE) imaging shows no contrast uptake from the mass (white star), typical for an RV thrombus

**Figure 4:**
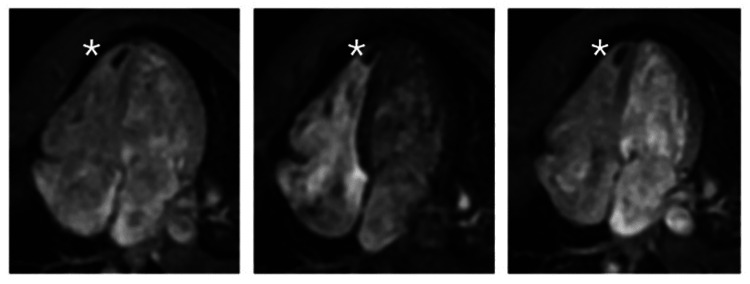
First-pass perfusion (FPP) imaging First-pass perfusion (FPP) imaging during the acute phase reveals an intracavitary low signal mass with no contrast uptake (white stars), indicative of a thrombus

TTE raised the suspicion of a thrombus, and we decided to investigate this further with a cardiovascular magnetic resonance (CMR) study. CMR revealed an LV of normal size and function [LV ejection fraction (EF): 54%, LV end-diastolic volume (EDV): 208 ml, LV end-systolic volume (ESV): 96 ml]. RV appeared to have a normal size with preserved systolic function [RVEF: 48%, RVEDV: 227 ml, RVESV: 118 ml, tricuspid annular plane systolic excursion (TAPSE): 24 mm]. Balanced steady-state free precession (bSSFP) cine images showed an oval-shaped mass of 16 x 6 mm within the cavity of the RV apex, with borders distinguishable from ventricular endothelium and trabeculations, raising the suspicion of an RV apical thrombus (Figure 3A). First-pass perfusion (FPP) was performed with the injection of a gadolinium contrast agent, which revealed in the RV an apical, avascular mass with low signal and no contrast uptake, findings indicative of a thrombus (Figure 4). Late gadolinium enhancement (LGE) sequences were performed with no contrast uptake of the mass, which is typical for an RV thrombus (Figure 3B).

**Figure 5:**
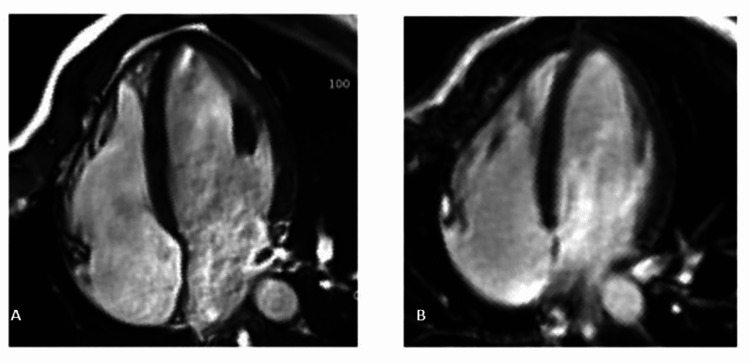
Cine balanced steady-state free precession (bSSFP) and late gadolinium enhancement (LGE) imaging after six months of follow-up 5A: Cine balanced steady-state free precession (bSSFP) imaging six months after anticoagulation treatment with no evidence of the RV thrombus 5B: Late gadolinium enhancement (LGE) imaging six months after anticoagulation treatment with full resolution of the RV thrombus

A hypercoagulable workup including antiphospholipid antibodies, antinuclear antibodies, beta-2-glycoprotein, anticardiolipin antibody, homocysteine, antithrombin, protein C, and protein S was normal. The recent COVID-19 infection appeared to be the only plausible cause of the RV apical thrombus. LMWH was bridged to warfarin. The patient was successfully discharged within the next few days after achieving therapeutic levels of international normalized ratio (INR) between 2 and 3. Following the anticoagulation of the patient for six months, a follow-up TTE (Figure 2B) showed a complete resolution of the RV thrombus. Similarly, a second CMR was performed, which showed no RV thrombus on bSSFP cine sequences or LGE sequences. A stack covering the RV from the base to the apex for both cine sequences and LGE sequences was used, showing no evidence of residual thrombus (Figures 5A, 5B).

## Discussion

Following the COVID-19 outbreak, a strong correlation between COVID-19 infection and hypercoagulopathy has been confirmed. This coagulopathy seems to be one of the most severe consequences of the disease. The COVID-19-related thrombosis as well as the abnormal coagulation parameters have been perceived as a poor prognostic marker in COVID-19 patients [2]. Among the most important predisposing factors for thrombosis in COVID-19 patients are hospitalization-related reduced mobility and prolonged immobilization, vascular inflammation, high levels of inflammatory markers, and direct virus-induced endothelial injury [1,2].

An autopsy study of patients who died from COVID-19 showed a high incidence of deep venous thrombosis (58%) and fatal pulmonary embolism (33%) [3]. Similarly, another study revealed that among patients with COVID-19 infection admitted to ICU, there is a higher incidence of pulmonary embolism (20.4%) [4]. There have already been cases in the literature describing transient right atrial thrombus in patients with COVID-19 infection [5,6], or mobile right ventricular thrombus [7] causing a pulmonary embolism; however, to our knowledge, this is the first case report involving in situ thrombosis of the RV, without pulmonary embolism, in a low-risk young patient with previous COVID-19 infection.

The prophylactic administration of low-molecular heparin in all patients with COVID-19 infection is of paramount importance. Similarly, early diagnosis of thrombosis is vital, and TTE might be a reasonable option in most patients with a history of COVID-19 infection. TTE remains the main imaging technique for assessing the RV function, but since thrombus and myocardium may have similar echogenicity, TTE alone is sometimes not suitable to differentiate these two types of tissue. Whenever there is a high suspicion of RV thrombus or in cases of an inconclusive TTE and/or inconclusive transoesophageal echocardiogram (TOE), advanced imaging techniques like CMR could be employed as an additional modality to confirm the diagnosis.

The superiority of CMR to detect thrombus in the RV with its higher sensitivity and specificity compared to the current standard with TTE and/or TOE has already been described in some case reports and case series [8,9]. Barbagallo et al. have described three cases with similar diagnostic ambiguity in the diagnosis of possible RV thrombus. In all three cases, the use of CMR modalities (especially FFP imaging and LGE sequences) was crucial for the final diagnosis and choosing the suitable anticoagulant therapy [8]. Tsang et al. presented a case of RV thrombus associated with an RV infarct secondary to a proximal right coronary artery thrombotic occlusion. In this case, RV thrombus was not evident on TTE but detected on both LGE CMR images and microsphere contrast TOE [9].

Irrespective of the cause of RV thrombus formation, multimodality imaging, especially the use of CMR, despite its cost, offers a very useful additional diagnostic tool. CMR can be a supplemental tool besides TTE/TOE in the diagnosis and follow-up of incidental intracardiac masses in the RV. In our study, the TTE findings raised the suspicion of a thrombus. RV thrombus was diagnosed after the CMR during the analysis of all the obtained images, i.e., bSSFP cine sequences, FPP, and LGE sequences.

## Conclusions

Hypercoagulability is a major complication of COVID-19 and thrombosis can occur both in arterial and venous circulation including the RV. These thrombotic events appear to be most common in hospitalized COVID-19 patients, but our case showed that thrombotic events can be present in low-risk post-COVID-19 patients as well. The recognition of intracardiac thrombi even in low-risk patients with a history of COVID-19 infection, like our case, and the immediate initiation of antithrombotic treatment to minimize the risk of embolization, is crucial. The use of advanced imaging techniques like CMR can help in confirming the diagnosis.
